# Supplementary material for: Use of complete medication history to identify and correct transitions-of-care medication errors at psychiatric hospital admission
Source: PLoS One. 2023 Jan 25;18(1):e0279903. doi: 10.1371/journal.pone.0279903 (PMC9876239; doi:10.1371/journal.pone.0279903)
Supplement: S1 Appendix — Data table describing the source of specific data elements and their formats to be included in the medication error collection form. (DOCX) [file pone.0279903.s001.docx]

| **Step** | **Data Point** | **Source – Admissions** | **Source - Outpatients** | **Outcome Format** |
| --- | --- | --- | --- | --- |
| **1** | **Patient MRN** | Password-protected Excel Spreadsheet | Password-protected Excel Spreadsheet | XXXXXX |
| **2** | **Date of Case** | Password-protected Excel Spreadsheet | Password-protected Excel Spreadsheet | MM/DD/YYYY |
| 3 | Admission Date | Epic > patient station > search MRN > choose admission encounter that includes case date | Epic > patient station > search MRN > identify there is no admission during case date | MM/DD/YYYY or N/A |
| 4 | Admission Day of Week | Calendar | N/A | Su/Mo/Tu/We/Th/ Fr/Sa or N/A |
| 5 | Seen at McLean PTA (Prior Year) | Identify any inpatient encounters at McLean in prior year | Identify any inpatient encounters at McLean in prior year | Y/N |
| 6 | Discharge Diagnosis | Highlight encounter > view principal problem in discharge summary | N/A | Copy & paste principle problem or N/A |
| 7 | Length of Stay | Upper right-hand corner of discharge summary | N/A | # of days or N/A |
| 8 | Age (Date of Case) | Patient header – adjusted for date of story as needed | Patient header – adjusted for date of story as needed | Years |
| 9 | Sex | Patient header | Patient header | M/F |
| 10 |  | Open encounter that includes story date (pharmacist) or select “full registration” and most recent encounter (technician) | Open encounter that includes story date (pharmacist) or select “full registration”> most recent encounter > first tab with patients name ONLY (technician) |  |
| 11 | Race | Demographics activity (pharmacist) or select “full registration” and most recent encounter (technician) | Demographics tab in Patient Station (pharmacist) or Full Registration in patient station (also check out education status – assess difference between note and this section) (tech) | Hispanic  Non-Hispanic  Unknown |
| 12 | Marital Status |  |  | Married  Single  Divorced  Widowed  Unknown  Other |
| 13 |  | Go to notes activity (pharmacist) or select chart review then notes (technician) | - |  |
| 14 |  | Select H&P note | - |  |
| 15 | Number of Active Medical Problems | H&P > Active Medical Problems | Patient Station > chart review > problem list | # |
| 16 | Total Number PTA Meds | H&P > Prior to Admission Medications | Chart review tab in Patient Station > Encounters tab > note from date of case (or closest PACT note) > medications section | #. If a prn and standing of same medication, count as 2 medications (list the same drug name twice in list). If two separate orders for the same drug, both standing, count as 1 medication. |
| 17 | List PTA meds |  |  | Drug names only, generic names except for combination drugs |
| 18 |  | Select Psych admission note | - |  |
| 19 | Admitting Diagnosis | Psych admission note > Provisional Diagnoses | - | Copy and paste. N/A for Outpatients/PACT |
| 20 | PACT Diagnosis | - | Note from date of case > ID/CC section | Copy and paste. N/A for Admissions |
| 20 | Location of Residence | Psych Admission Note > HPI/Social History | Note from date of case > Rehabilitation services plan OR Support/Psychosocial Services OR Additional services plan section | Home/ assisted living/ group home/ nursing home/ unknown/ other |
| 21 | Transfer from ED/OSH | Psych Admission Note > HPI | N/A | Y/N or N/A |
| 22 | Employment | Psych Admission Note > HPI/Social History | Note from date of case > Rehabilitation services plan OR Support/Psychosocial Services OR Additional services plan section | Y/N or unknown  (for the purposes of data collection, “on disability” is N) |
| 23 | Highest Level of Education | Psych Admission Note > HPI/Social History | Note from date of case > Rehabilitation services plan OR Support/Psychosocial Services OR Additional services plan section | Below high school/ high school diploma/ some college/ college educated/ unknown |
| 24 | Arrival Time | Summary > ADT events > arrival at MCL Clinic Eval Ctr | N/A | HH:MM, military time or N/A |

Please list all PTA medications within the box below:
