# Supplementary material for: Use of complete medication history to identify and correct transitions-of-care medication errors at psychiatric hospital admission
Source: PLoS One. 2023 Jan 25;18(1):e0279903. doi: 10.1371/journal.pone.0279903 (PMC9876239; doi:10.1371/journal.pone.0279903)
Supplement: Attachment — Submitted filename: Response to Reviewers.docx [file pone.0279903.s004.docx]

To our reviewers and editors at PLOS One, thank you for your constructive comments. Please find our responses to each comment below:

- We have included an Ethics Statement in the Methods section of our manuscript at the direction of the editor.
- We have added additional clarification to our financial disclosure to our cover letter at the direction of the editor.
- We have included our updated Data Availability Statement in our cover letter. Also, we have uploaded our Minimum Underlying Dataset as Supporting Information and will include the following statement in our manuscript: All data is included in the manuscript and/or Supporting Information. We added legends for all Supporting Information and named the files to be consistent with PLOS’ submission guidelines.
- Instead of breaking out the Abstract into separate Objective, Methods, Results, and Conclusions, we have amended the manuscript to include the Abstract in one cohesive paragraph.
- We double checked to ensure that none of the papers we cited have been retracted. All our citations correctly refer to the preceding statements.

Reviewer Comments Addressed:

- We have provided a sentence at the beginning of the methods section to provide more context on the nature of the hospital.
- We have clarified what a “legitimate medication discrepancy” is in the methods section by removing the word “legitimate” and describing a medication discrepancy.
- We have added a brief summary of the role of a TOC pharmacist in the context of transitions of care and supporting citations for the benefits of this clinical workflow.
- We have moved the Medication Error Type of “Other” to the bottom of the list instead of the middle where it makes more sense to display.
- We explored the 2^nd^ most common medication error we found (Omission) in the discussion as requested.
- We agree that the methodology for scoring medication that have multiple uses belongs in the methods section and has been moved there.
- We have added references that support our claims of the difficulty to quantify the scale and severity of medication errors.
- We deleted the second repetitive sentence identified in the conclusion.
- In support of our claim that we “proved the value of pharmacist expertise while utilizing a medication history tool for TOC medication reconciliation,” we have added references and clarification adjacent this claim, changed the word ‘proved’ to ‘support’, and we also introduced this concept with this sentence earlier in the discussion “Pharmacists are more in tune to the miniscule differences between medication formulations and dosages”
- We added the following clarifying sentence to address how a follow up study measuring medication cost may add to the omission findings presented “These were not directly addressed by TOC pharmacy staff during the time setting of this study and would be an interesting follow up analysis to measure the effect of prescription drug costs on omission medication errors in psychiatric patients.”
- We have included citations to support the claim “oftentimes, patient have inaccurate accounts of their medication history, current medication list, or refill history” in the discussion.

Font size

- Standard font was used in the manuscript
- Major heading were formatted In bold 18pt font
- Sub heading were formatted in bold 16pt font

Table formats

- Table title and caption was inserted after paragraph of initial mention
- Actual table itself was inserted after paragraph of initial mention
- Tables were converted into editable cells
- 1pt font was used for border
- 3pt font was used for lever
- Only black color font was used

Any other changes, etc.

- Figure title and caption was inserted after paragraph of initial mention
- Figure is provided separately
- Acknowledgements were added before references
- Supporting files are added after references
- References are recited into correct format
- Retractable references checked (none noted)

We hope that these revisions satisfy the reviews and eagerly await your decision.

Sincerely,

Carol Aboud, PharmD, RPh

Chief Pharmacy Officer

McLean Hospital – Mass General Brigham

115 Mill St., Belmont, MA 02478

(phone) 617.855.2465 | (fax) 617.855.3355

caboud@partners.org

caboud@mclean.harvard.edu

www.mcleanhospital.org
