# Supplementary material for: Use of complete medication history to identify and correct transitions-of-care medication errors at psychiatric hospital admission
Source: PLoS One. 2023 Jan 25;18(1):e0279903. doi: 10.1371/journal.pone.0279903 (PMC9876239; doi:10.1371/journal.pone.0279903)
Supplement: Attachment — Submitted filename: Response to Reviewers.docx [file pone.0279903.s005.docx]

To our reviewers and editors at PLOS One, thank you for your constructive comments. We are happy to accommodate your additional revisions to improve the readability of our manuscript. Please find our responses to each comment below:

Reviewer Comments Addressed:

Abstract

- We have edited the abstract to eliminate long sentences to improve the readability of the paper.

.
Introduction

- We have removed the term feasible and feasibility from the manuscript and re-worded our explanations to reflect “assessing the benefit” of our methodology in response to our reviewer’s comments.

Methods:

- We edited the following sections to improve reading clarity: Inclusion Criteria, Medication Error Data Collection, and Medication Error Scoring Methodology.

Results

- We added a space between the table and the next paragraph for lines 216-217.

Discussion

- We significantly edited this section by shortening sentences and breaking out an additional section to improve readability.

Figure:

- We did a full re-design of the figure ‘DrFirst Medication Reconciliation Intervention Study Timeline’ per the reviewer’s suggestions.

Thank you for the clarifying reviews. We believe they will greatly enhance the readability of our manuscript and eagerly await your response.

Sincerely,

Carol Aboud, PharmD, RPh

Chief Pharmacy Officer

McLean Hospital – Mass General Brigham

115 Mill St., Belmont, MA 02478

(phone) 617.855.2465 | (fax) 617.855.3355

caboud@partners.org

caboud@mclean.harvard.edu

www.mcleanhospital.org
